# The Alteration of Nasopharyngeal and Oropharyngeal Microbiota in Children with MPP and Non-MPP

**DOI:** 10.3390/genes8120380

**Published:** 2017-12-11

**Authors:** Zhiwei Lu, Wenkui Dai, Yanhong Liu, Qian Zhou, Heping Wang, Dongfang Li, Zhenyu Yang, Yinhu Li, Gan Xie, Shuaicheng Li, Yuejie Zheng

**Affiliations:** 1 Department of Respiratory Diseases, Shenzhen Children’s Hospital, Shenzhen 518026, China; luzhiwei1950@163.com (Z.L.); szetgmy@163.com (H.W.); xiegan1987@163.com (G.X.); 2 Department of Computer Science, City University of Hong Kong, Hong Kong 999077, China; daiwenkui84@gmail.com; 3 Department of Microbial Research, WeHealthGene Institute, Shenzhen 518129, China; liuyanhong@wehealthgene.com (Y.L.); zhouqian@wehealthgene.com (Q.Z.); lidf@wehealthgene.com (D.L.); yangzhy@wehealthgene.com (Z.Y.); liyh@wehealthgene.com (Yin.L.); 4 Institute of Statistics, NanKai University, Tianjin 300071, China

**Keywords:** 16S rDNA, microbiota, *Mycoplasma pneumoniae*, nasopharyngeal, oropharyngeal

## Abstract

**Background:** In recent years, the morbidity of *Mycoplasma pneumoniae* pneumonia (MPP) has increased significantly in China. A growing number of studies indicate that imbalanced respiratory microbiota is associated with various respiratory diseases. **Methods:** We enrolled 119 children, including 60 pneumonia patients and 59 healthy children. Nasopharyngeal (NP) and oropharyngeal (OP) sampling was performed for *16S ribosomal RNA (16S rRNA)* gene analysis of all children. Sputum and OP swabs were obtained from patients for pathogen detection. **Results:** Both the NP and OP microbiota of patients differ significantly from that of healthy children. Diseased children harbor lower microbial diversity and a simpler co-occurrence network in NP and OP. In pneumonia patients, NP and OP microbiota showed greater similarities between each other, suggesting transmission of NP microbiota to the OP. Aside from clinically detected pathogens, NP and OP microbiota analysis has also identified possible pathogens in seven cases with unknown infections. **Conclusion:** NP and OP microbiota in MPP and non-MPP are definitely similar. Respiratory infection generates imbalanced NP microbiota, which has the potential to transmit to OP. Microbiota analysis also promises to compliment the present means of detecting respiratory pathogens.

## 1. Introduction

Various pathogens, such as *M. pneumoniae*, *Streptococcus pneumoniae*, and respiratory syncytial virus (RSV), are common infectious agents of pneumonia, which is one of the principal causes of childhood morbidity and mortality [1,2,3,4]. Generalized clinical detection of pathogens was primarily based on bronchoalveolar lavage fluid (BALF) sampling, which was confined to pneumonia with severe diseases [5] and easily contaminated by respiratory flora in upper respiratory tracts (URTs) [6]. In addition, culturing and nucleic acid detection of specific pathogens make it difficult to discern infection within the carriage [7,8,9], given the asymptomatically colonized pathogens. 

Numerous reports demonstrated an imbalance of microbial flora in pneumonia patients who harbored lower diversity and richness of microbiota in URTs [2,5]. Moreover, this imbalanced respiratory microbiota pattern varied with infectious agents [10]. For instance, the *Moraxella lacunata*-dominated microbiota structure was identified in viral pneumonia [2], while the *S. pneumoniae*, *Haemophilus influenzae* complex or *Moraxella catarrhalis* dominated in the nasopharyngeal (NP) microbiota of bacterial pneumonia. Furthermore, the clustering of URTs microbiota in patients with pneumonia or bronchiolitis held the potential to predict disease severity, as a previous study demonstrated that the high relative abundance of either lactobacilli, *Rothia* or *S. pneumoniae*, was related to a high pneumonia severity index (PSI) [5].

Previous research indicated different NP and oropharyngeal (OP) microbiota between healthy and diseased children [11]. A two-year prospective study suggested that the transmission of imbalanced microbiota between the NP and OP of diseased children held the potential to further trigger lung infection [12]. Over the past few years, *M. pneumoniae* was a frequent cause of community-acquired pneumonia (CAP) and resulted in a high morbidity ratio in China [4]. In this study, we would like to answer two questions: (1) How does the NP and OP microbiota, and the microbiota transmission in URTs, of Chinese children with pneumonia differ from that of healthy children? (2) Are *Mycoplasma pneumoniae* pneumonia (MPP) and non-MPP infections associated with different NP and OP microbiota alterations?

## 2. Materials and Methods

### 2.1. Ethical Approval

This study was approved by the Ethical Committee of Shenzhen Children’s Hospital with registration number 2016013. All the guardians of recruited children provided their informed consent. All procedures performed in this study were in accordance with the ethical standards of the institutional and/or national research committee, as well as the 1964 Helsinki declaration and its later amendments or comparable ethical standards.

### 2.2. Diagnosis, Sampling and Pathogen Examination

Pneumonia was diagnosed through characteristic chest radiographic abnormalities that were consistent with pneumonia (Table 1). Patients were selected based on following criteria: no asthma; no respiratory infection; and no exposure to antibiotics for 1 month before sampling. Age-matched, healthy children were recruited after physical examination in Shenzhen Children’s Hospital according to the following criteria: no asthma or family history of allergy; no history of pneumonia; no wheezing, fever, cough, or other respiratory/allergic symptoms at sampling 1 month prior to the study; no antibiotic exposure for 1 month before the study; no disease symptoms within 1-week after sampling. NP (25-800-A-50, Puritan, Guilford, NC, USA) and OP (155C, COPAN, Murrieta, CA, USA) swabs were collectively frozen at −80 °C within 10 min of sampling. Unused swabs and opened swabs, which were shook in the air for 10 s, were selected as negative controls. 

Sputum was used for bacterial culturing, which was conducted to detect *Acinetobacter baumannii*, *H. influenzae*, *Haemophilus parainfluenzae*, *M. catarrhalis*, *Staphylococcus aureus*, *Staphylococcus haemolyticus*, and *S. pneumoniae* [13]. OP swabs were used to conduct nucleic acid testing (NAT). Adenovirus (AdV) and RSV were detected by a D3 Ultra DFA Respiratory Virus Screening & ID Kit (Diagnostic hybirds, Inc., Athens, OH, USA), while Epstein-bar virus (EBV), Cytomegalovirus (CMV), and Influenza A (H1N1) were detected by a EBV PCR Fluorescence quantitative diagnostic Kit, a Diagnostic Kit for Quantification of Human CMV DNA (PCR-fluorescence), and a Diagnostic kit for H1N1 RNA (PCR-fluorescence probing) (DaAnGene, Guangzhou, China, http://daan.joomcn.com/), respectively. *M. pneumoniae* and *Chlamydia pneumoniae* were diagnosed through a Diagnostic Kit for *M. pneumoniae* DNA (PCR-fluorescence probing) (DaAnGene, Guangzhou, China) and Anti *C. pneumoniae* ELISA (IgM) (EUROIMMUN AG, Lübeck, Germany). The Group A *Streptococcus* (GAS) detection was performed by Binax NOW^®^ Strep A Card (Alere Scarborough, Inc., Scarborough, ME, USA).

### 2.3. DNA Extraction, Library Construction, and Sequencing

The genomic DNA of microbiota was extracted from swab samples using the Power Soil DNA Isolation Kit (Mo Bio Laboratories, Carlsbad, CA, USA). The V3-V4 region of the *16S rRNA* gene [14] was amplified using a PCR kit (TransGenAP221-02, Beijing, China) and quantified by Qubit (Thermo Fisher, Singapore). The qualified libraries were then sequenced by Illumina MiSeq sequencing platform (Illumina, San Diego, CA, USA) [15]. All data have been deposited in GenBank under accession number: SRP090593 (healthy children) and SRP109319 (children with pneumonia).

### 2.4. Bioinformatics Analysis and Visualization

Data filtration, taxonomic classification, and diversity calculation were conducted following our previous study [16]. PERMANOVA was applied to evaluate the confounding factors of microbiota composition and species-level classification of common pathogens referred to as the Live Tree Project (LTP) [17,18]. The inter-group comparison was conducted by the Wilcoxon rank-sum test, and the significance of multiple comparisons was adjusted by FDR. The distance between NP and OP microbiota was calculated by Bray-Curtis dissimilarity. The correlation of components with relative abundance—≥0.1% in microbiota—was assessed by Spearman’s rank correlation coefficient, with parameter coefficient ≥0.6 and *p*-value ≤0.05. Data visualization was produced by package ‘ggplot2’ of R software (v3.2.3) and Cytoscape (v3.4.0).

## 3. Results

### 3.1. Sample Information, Data Output, and Confounder Analysis

In this study, we enrolled 119 children, including 60 pneumonia inpatients and 59 age-matched healthy children (Table 1). According to the clinical detection, 29 cases were infected with *M. pneumoniae*, while 31 were diagnosed as infected by other or unknown pathogens (Table 1). None of the inpatients were admitted to the pediatric intensive care unit (PICU) or given mechanical ventilation during hospitalization. Most of inpatients remained in the hospital for less than 3 weeks but with one exception: P-33, who suffered the longest hospitalization time (37 days) (Table S1).

A total of 9,141,686 high-quality tags were produced, averaging 37,986 (22,208~46,854), 28,669 (15,288~41,626), 42,972 (25,465~47,409), and 43,844 (29,453~47,473) for the NP-health (NP-H), OP-health (OP-H), NP-pneumonia (NP-P), and OP-pneumonia (OP-P) group, respectively. Association analysis indicates that pneumonia onset is the most significant factor (*p*-value < 0.001) (Table S2) in explaining variations in microbial samples. 

### 3.2. OP and NP Microbiota of Patients Differ Significantly from that of Healthy Children

**Figure 1:**
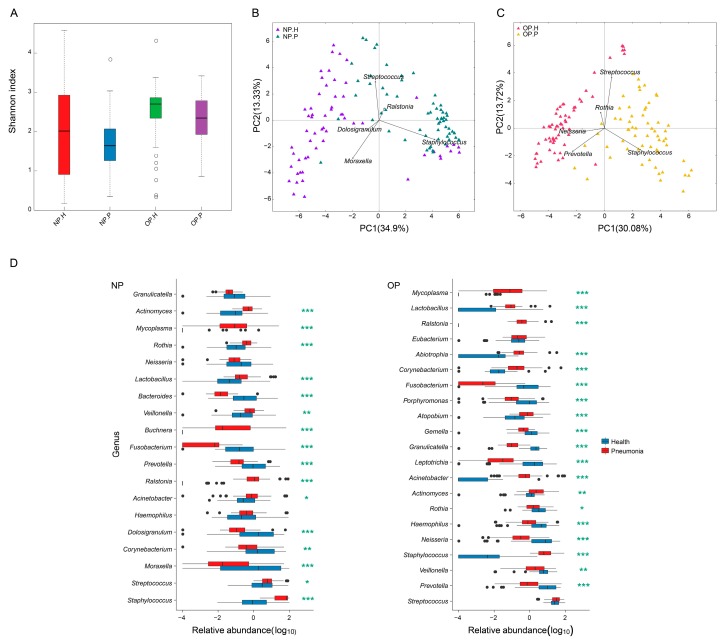
Nasopharyngeal (NP)/ oropharyngeal (OP) microbiota structure in pneumonia patients and healthy children. (**A**) Shannon index of NP and OP microbiota in patients and healthy infants; (**B**) principal components analysis (PCA) of NP samples (purple triangles stand for healthy controls, and dark green triangles stand for pneumonia subjects); (**C**) principal components analysis (PCA) of OP samples (red triangles stand for healthy controls, and yellow triangles stand for pneumonia subjects); (**D**) comparison of dominated genera between NP/OP microbiota of patients and that of healthy infants. Vertical axis represents genus name, and horizontal axis shows the log10 value of relative abundance. *, ** and *** represent *q*-value ≤ 0.05, ≤0.01, and ≤0.001, respectively.

Generally, bacterial diversity in NP is lower than in that of OP for all children, while the diversity of NP and OP microbiota in patients is also lower than in that of healthy children (Figure 1A). NP and OP microbiota analysis could clearly separate healthy and diseased samples (Figure 1B,C).

Firmicutes predominates, both in OP and NP microbiota of all children, but accumulates significantly in pneumonia patients (OP: 63.55% vs. 47.05% in healthy children, *q*-value < 0.001; NP: 72.09% vs. 43.06% in healthy children, *q*-value < 0.001) (Table S3). By contrast, Bacteroidetes are enriched in the OP and NP microbiota of healthy children (OP: 19.64% vs. 5.34%, *q*-value < 0.001; NP: 7.54% vs. 0.84%, *q*-value < 0.001) (Table S3). Proteobacteria accounts for a lower proportion of NP and OP microbiota in patients in comparison with healthy children, although this does not have statistical significance.

At genus-level, *Mycoplasma*, *Staphylococcus*, *Lactobacillus*, *Ralstonia*, *Acinetobacter*, and *Actinomyces* amass significantly in both the NP and OP microbiota of inpatients, while *Prevotella* is particularly enriched in healthy children (Figure 1D, Table S4). *Streptococcus* prevails in NP-P microbiota (Figure 1D, Table S4), while *Moraxella* and *Dolosigranulum* accumulate in NP-H microbiota (Figure 1D, Table S4). *Haemophilus* accounts for a large proportion of OP-H microbiota, while *Corynebacterium* similarly enriches OP-P microbiota (Figure 1D, Table S4).

**Figure 2:**
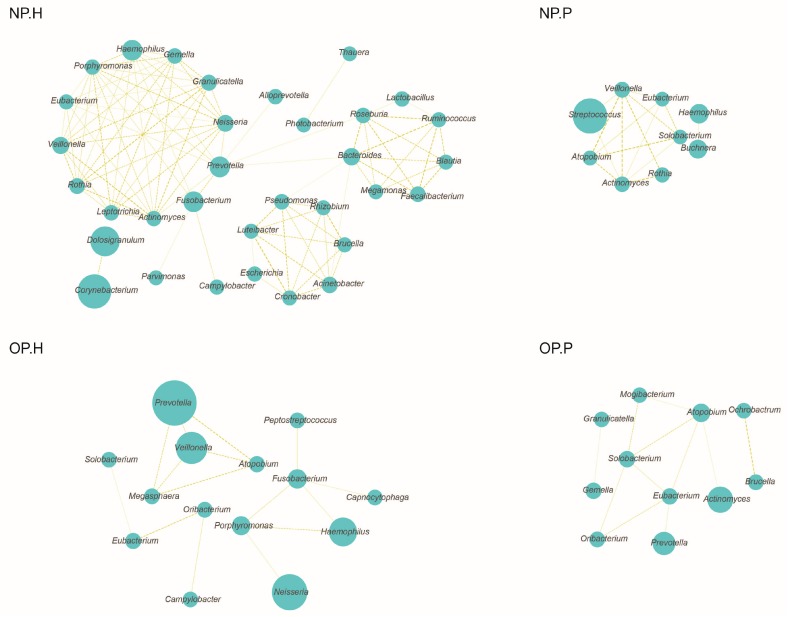
Co-occurrence network of NP/OP microbiota in pneumonia patients and healthy children. The circle size represents the relative abundance, and the density of the dashed line represents the Spearman coefficient.

The concurrence bacterial network (Figure 2) of NP-H is comprised of 33 genera featured by three core nodes, namely the *Prevotella*, *Roseburia*, and *Bacteroides*. Nevertheless, only 9 genera are included in bacterial network of the NP-P group. Identical with NP microbiota, the OP microbiota of diseased children also features a simpler concurrent network when compared to healthy children (Figure 2).

### 3.3. NP and OP Microbiota Pattern of Inpatients Have an Increased Similarity to Each Other

**Figure 3:**
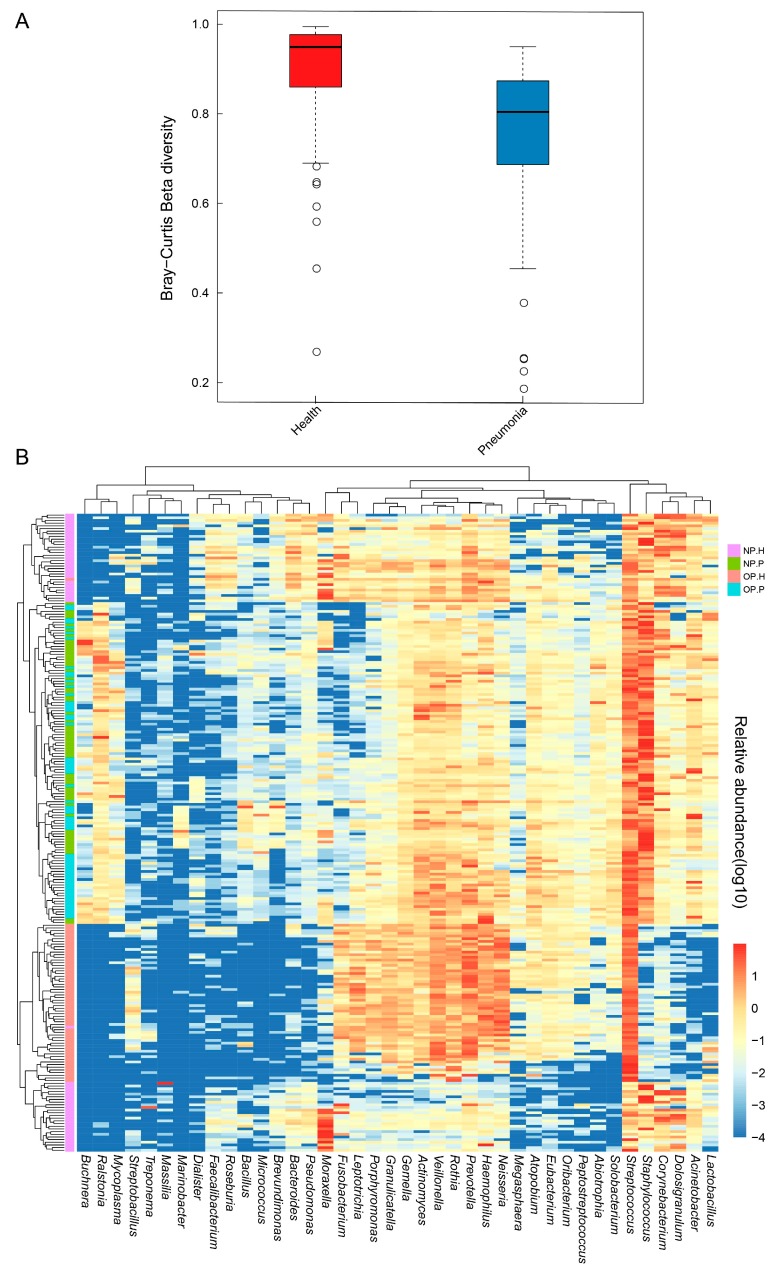
Dissimilarity and clustering of NP and OP samples. (**A**) Divergence between NP and OP microbiota based on Bray-Curtis dissimilarity; (**B**) the Log10 value of relative abundance was proportional to the color, from blue to red.

Compared to healthy children, the dissimilarity of NP and OP microbiota in patients is considerably smaller (Figure 3A). Further analysis indicates that all samples can be clustered into four main groups (Figure 3B). NP and OP samples of diseased children are contained in the same cluster. By contrast, OP samples in healthy children are contained in a cluster, with the exception of H-49. NP samples in healthy children are separated into two clusters, except for the sample H-8, which is assigned to the OP cluster of healthy children.

Apart from the enrichment of clinically diagnosed pathogens Mycoplasma and Staphylococcus in both the NP-P and OP-P microbiota of patients, we also identified the OP-P microbiota accumulation of Corynebacterium, which primarily dominates in NP-H microbiota.

### 3.4. Microbiota Analysis Can Be Complementary to Clinical Detection of Respiratory Pathogens

**Figure 4:**
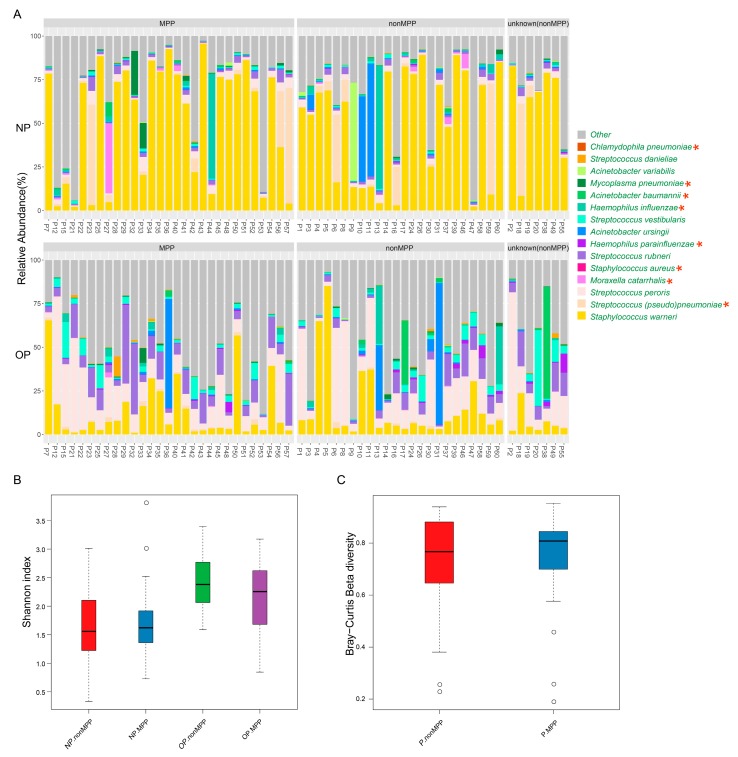
Microbiota comparison between *Mycoplasma pneumoniae* pneumonia (MPP) and non-MPP group. (**A**) Histogram based on species-level of common pathogens in NP/OP. Samples in MPP, non-MPP, and unknown (non-MPP) are ranked by age, respectively, from younger to the older. * stand for pathogens, which were also detected by conventional method; (**B**) Shannon index of NP/OP microbiota in MPP and non-MPP; (**C**) distance between NP and OP samples in MPP and non-MPP patients based on Bray-Curtis dissimilarity.

Clinical detection has indicated *M. pneumoniae* infection in 29 cases (MPP group), non-*M. pneumoniae* infection in 24 cases (non-MPP group), and an unknown pathogen infection in 7 cases (unknown (non-MPP) group) (Figure 4A, Table S5). The incidence of mixed infection in MPP is 21/29, while it is 8/24 in the non-MPP group. The hospitalization time in MPP, non-MPP, and unknown (non-MPP) averages at 10.4, 9.5, and 7.4 days, respectively. In addition, the MPP group has the highest proportion of fever (72.41%) and abnormal CRP (75.86%).The microbiota diversity of NP/OP differs slightly between MPP and the two other groups (Figure 4B). Moreover, the NP-OP microbiota’s dissimilarity within the MPP group is also similar to that of non-MPP groups (Figure 4C).

*Mycoplasma* species show significant accumulation in pneumonia patients, with no statistical difference in NP/OP microbiota between MPP and non-MPP groups (Table S6). In addition to *M. pneumoniae*, four other *Mycoplasma* species are also identified (Tables S6 and S7), including *Mycoplasma faucium*, *Mycoplasma salivarium*, *Mycoplasma sualvi*, and *Mycoplasma testudinis*. *M. pneumoniae* is the dominant species of *Mycoplasma* in NP (26/29 in MPP, 19/24 in non-MPP, and 5/6 in the unknown (non-MPP) group) and OP (25/29 in MPP, 19/24 in non-MPP, and 5/6 in unknown (non-MPP) group) of patients (Table S7), followed by *M. salivarium*. For P-33, who suffered the longest hospitalization period (37 days) (Table S1), a high abundance of *M. pneumoniae* was identified both in NP and OP microbiota, with proportions of 14.51% and 8.48%, respectively (Figure 4A, Table S5). 

*Staphylococcus warneri* is dominant in the NP microbiota of six cases, where the patient was diagnosed with an unknown pathogen infection. *S. (pseudo)pneumoniae* accounted for 52.83% of OP microbiota in P-18 (Figure 4A, Table S5), who had no fever and suffered 17 days hospitalization. In OP microbiota, P-38 possessed 63.67% of *A. baumannii* and P-55 consisted of 10.88% *H. parainfluenzae* (Figure 4A, Table S5). *Streptococcus peroris* represented 79.17% of OP microbiota for P-2 (Figure 4A, Table S5), who had a seven day fever during hospitalization. For 24 other patients, NP/OP microbiota analysis results conformed with those of a clinical diagnosis for *S. pneumoniae* (Figure 4A, Table S5), while *M. catarrhalis* constituted a low proportion of microbiota, even for patients who were diagnosed with a *M. catarrhalis* infection.

## 4. Discussion

Our study shows a simpler NP and OP microbiota structure and co-occurrence network in children with pneumonia who were diagnosed with MPP or non-MPP. The level of respiratory commensals decreased after infection, such as a reduced number of lactic acid-producing bacterium *Dolosigranulum* [19]. A previous report also indicated decreased levels of *Prevotella* after a respiratory infection, which antagonized the LPS produced by *H. influenzae* and inhibited the Toll-like receptor 4 (TLR4) mediated mucosal inflammation [20]. Respiratory pathogens intrude greatly on the respiratory tract through multiple virulence factors [21,22,23,24] and evade the immune response of the host [25,26], which can induce a proliferation of pathogens and may explain the simpler NP and OP microbiota structure of patients [16,27,28].

In addition, we observe a higher similarity between NP and OP microbiota in patients, which can be explained by a robust intrusion of pathogens through the nasal cavity and the transmission from NP to OP and lung [29,30]. Microbes in the air are transmitted to the lung through the nasal cavity and NP [31], which affect the initial infection at NP and subsequent transmission. In parallel, we found OP-P enrichment of Corynebacterium, which was dominant in the NP microbiota of healthy children.

Compared to the non-MPP patients, MPP cases suffered a longer hospitalization time, a higher rate of abnormal CRP values, and fever. This may be related to the robust pathogenicity of *M. pneumoniae*, such as cytoadherence to respiratory epithelium and cytotoxicity release [32]. Meanwhile, the MPP group has a higher incidence of mixed infection with viruses or bacteria than non-MPP patients. The high ratio of mixed infection in our study is inconsistent with the previous report [33], which implied that the practice for CAP should be country- or region-specific.

Conventional pathogen detection is limited to known typical pathogens [34,35] and emerging reports demonstrating that the 16S/metagenomics method could assist in respiratory pathogen detection [36,37,38]. In a prior report, gram staining and culturing indicated that *S. aureus* and *Streptococcus anginosus* gave rise to infections in the lung, yet targeted therapy was not effective [37]. However, microbiota analysis showed that the patient had achieved significant remission from the *Streptococcus intermedius* infection after 14 days targeted therapy [37]. Several reports indicated the potential of metagenomic sequencing in diagnosing respiratory pathogens in ventilation associated pneumonia (VAP) and hematopoietic cellular transplant (HCT) patients [36,38]. This study shows that microbiota analysis is complementary to the present means of detecting *M. pneumoniae*, such as enrichment of other *Mycoplasma* species, for example, *M. testudinis*. In addition to *Mycoplasma*, 16S rDNA analysis also provides clues regarding other infectious pathogens affecting seven patients with a clinically unknown infection, including a >50% proportion of *A. baumannii* and *Streptococcus peroris* in the OP microbiota of P-38 and P-2, respectively.

Several limitations affecting this study should be noted. The first is the small number of recruited cases and lack of microbiota analysis in sputum/lung samples, which made it difficult to clearly comprehend the microbial transmission mode, as well as the significance of NP/OP microbiota analysis. Although the pneumonia samples were divided into MPP and non-MPP groups, we could not discover group-specific patterns to deepen our understanding of infection-related dysbiosis. The 16S rDNA analysis cannot identify species-level pathogens definitively, nor explain functional dysbiosis in infection-caused microbiota imbalance.

## 5. Conclusions

This study provides insight into imbalanced URTs microbiota in Chinese MPP and non-MPP children. Profiling possible pathogens through microbiota analysis also holds the potential to complement present means of pathogen detection.

## Figures and Tables

**Table 1:** Sample information.

	Healthy Children (*n* = 59)	Pneumonia Patients (*n* = 60)
**Characteristics**		
Gender		
Female	33	19
Male	26	41
Age (years)	2.8 (0.1–9.9)	2.8 (0.2–12.7)
Delivery Mode		
Caesarean section	20	22
Vaginally born	39	38
Feed Pattern		
Breast feed	18	40
Breast feed + Milk feed	31	6
Milk feed	10	14
Family history of allergy	-	1
History of pneumonia	-	12
Asthma	-	-
**Clinical records**		
Lung consolidation, atelectasis, infiltration	NA	60
Hospitalization time (days)	-	9 (2–37)
Fever	-	26
Cough	-	57
Wheezing	-	16
CRP (<0.499 mg/L)	NA	21
PCT (<0.5 ng/mL)	NA	60
Eosinophil (0.5–5%)	NA	33

“-” represents no detected; “NA” represents not available; CRP, C-response protein; PCT, Procalcitonin.

## Supplementary Materials

The following are available online at www.mdpi.com/2073-4425/8/12/380/s1. Table S1: Sample information of pneumonia patients and healthy children. Table S2: Evaluation of factors’ contribution by PERMANOVA. Table S3: Wilcoxon rank-sum test result of NP and OP at phylum level between healthy children and pneumonia patients. Table S4: Wilcoxon rank-sum test result of NP and OP at genus level between healthy children and pneumonia patients. Table S5: Species-level detection between conventional and sequencing method. Table S6: Comparison of *Mycoplasma* species in NP/OP among MPP, non-MPP, and healthy group. Table S7: Distribution of *Mycoplasma* species in NP and OP of patients and healthy children.
